# Splicing-driven post-translational dysregulation: a new frontier for precision cancer medicine and immunotherapy

**DOI:** 10.1007/s12094-025-04217-0

**Published:** 2026-01-24

**Authors:** Sael Alatawi

**Affiliations:** 1 Department of Medical Laboratory Technology, Faculty of Applied Medical Sciences, University of Tabuk, 47512 Tabuk, Saudi Arabia; 2 Institute of Cancer and Genomic Sciences, University of Birmingham, Birmingham, UK

**Keywords:** Splicing factor mutations, Post-translational modifications, Spliceosome, Immune evasion, Precision oncology, Therapeutic targeting

## Abstract

Cancer is a disease marked by widespread molecular dysregulation, including alterations in gene expression, signaling pathways, and protein function. Among the critical regulators of protein function are post-translational modifications (PTMs), which fine-tune protein stability, activity, localization, and interactions. At the same time, more and more data has shown that mutations in parts of the splicing machinery, such as *SF3B1*, *SRSF2*, *U2AF1*, and *ZRSR2*, are common causes of different types of hematologic and solid tumors. Although the transcriptome implications of these mutations have been thoroughly delineated, their subsequent impacts on PTM regulation are still predominantly unexamined. This review seeks to address this deficiency by emphasizing the nascent connections between spliceosome mutations and the alteration of PTM landscapes in cancer. We suggest that modified splicing of PTM-related enzymes and substrates could significantly transform the cancer proteome, providing novel mechanistic insights and therapeutic prospects. We also look into how splicing-driven PTM changes, especially those that affect ubiquitination pathways and other important modification systems, affect the immune landscape of tumors. This gives us new information about how tumors with splicing mutations become more fit by changing the pathways that control the immune system and tumor surveillance.

## Introduction

RNA splicing is the process of taking intronic regions out of precursor mRNA and carefully assembling exons to make mature transcripts. This is important for making a variety of protein isoforms while preserving the stability and coding capability of mRNA. [1]. In the past few years, it has been discovered that frequent somatic mutations in spliceosomal components constitute significant events in many cancers. [2], particularly hematologic cancers [3]. These mutations not only change normal splicing patterns, but they also make abnormal protein isoforms that add more fitness to cancer cells [4, 5]. Recognizing the frequency, functional implications, and clinical significance of key splicing factor mutations establishes a basis for investigating their subsequent impacts on cellular mechanisms, especially post-translational modification networks that regulate protein function and maintain cellular homeostasis. A systematic characterization of intronic mis-splicing mutations in human cancers has demonstrated their extensive influence on tumor biology [6]. The rewiring of alternative splicing is a key feature of cancer biology, leading to oncogenic transformation through various mechanisms [7]. Notably, recent work has highlighted that broad splicing dysregulation, even in the absence of specific mutations, can serve as a therapeutic target in heterogeneous leukemias [8].

### Prevalence and recurrent patterns of splicing factor mutations

Somatic mutations in essential components of the RNA splicing machinery especially in genes like *SF3B1*, *SRSF2*, *U2AF1*, and *ZRSR2* have been identified as a common characteristic in multiple human malignancies, highlighting the vital importance of splicing regulation in carcinogenesis [9, 10]. The incidence and patterns of these mutations differ markedly among cancer types, indicating tissue-specific susceptibilities to spliceosomal disruption. Table 1 gives a list of important splicing factor mutations in many types of cancer. It shows the most significant genes and their linked features. In solid tumors, mutations in splicing factors happen less often and are usually only found in certain subtypes. For instance, *SF3B1* mutations are present in about 15–20% of uveal melanomas, but *RBM10* mutations are more prevalent than other splicing factors in lung adenocarcinoma. In the majority of epithelial malignancies, these mutations are either infrequent or their relevance remains ambiguous, indicating that solid tumors may employ different methods to attain similar oncogenic effects [11]. In contrast, hematological malignancies exhibit a significantly elevated frequency of splicing factor mutations, especially in myeloid lineage malignancies.

**Table 1:** Key splicing factor mutations in cancer

Gene	Component Type	Common Mutations	Highest Frequency Cancer	Frequency (%)	Cancer Association	References
*SF3B1*	Core (U2 snRNP)	K700E, H662Q, R625C	MDS	20–25	Strongly associated with MDS, particularly ring sideroblast phenotype; promotes favorable prognosis but linked to genomic instability in CLL; common in uveal melanoma (15–20%)	[83, 84]
*SRSF2*	Regulatory (SR protein)	P95H/L/R	CMML	40–50	Frequent in CMML and high-risk MDS; associated with unfavorable prognosis and progression to secondary AML; alters EZH2 splicing affecting chromatin regulation	[85]
*U2AF1*	Core (U2 snRNP)	S34F/Y, Q157R/P	MDS	8–12	Common in MDS and AML; associated with adverse prognosis; modifies splicing of DNA repair genes (RAD51) and immune-related genes; linked to impaired neutrophil function	[86, 87]
*ZRSR2*	Core (U12 snRNP)	Nonsense, frameshift	CMML	10–15	Recurrent in CMML and AML; loss-of-function mutations; affects minor spliceosome function; associated with poor prognosis	[88]
*RBM10*	RNA binding	Nonsense, frameshift	Lung adenocarcinoma	8–12	Most common splicing factor mutation in lung adenocarcinoma; tumor suppressor-like mutations; rare in hematologic malignancies	[89]
*PRPF8*	Core (U5 snRNP)	Point mutations	MDS	1–2	Infrequent in MDS; affects spliceosome assembly and function; associated with splicing dysregulation	[90]
*RBM39*	RNA binding	Point mutations	Pan-cancer	2–4	Identified across multiple cancer types; involved in splicing regulation and protein synthesis; target of aryl sulfonamide compounds	[91]
*SRSF1*	Regulatory (SR protein)	Overexpression	Lung cancer	5–8	Upregulated in lung cancer; promotes oncogenic splicing patterns; associated with poor prognosis	[2]
*SF1*	Core (U2 snRNP)	Point mutations	Pan-cancer	1–3	Recently identified as driver in multiple cancer types; involved in 3' splice site recognition; interacts with U2AF2 at the spliceosome; affects splicing fidelity across multiple genes	[11, 92]
*U2AF2 (U2AF35)*	Core (U2 snRNP)	E393D, other point mutations	MDS/AML	2–5	Mutations in MDS and AML; works with U2AF1 in 3' splice site recognition; E393D mutation found in AML, MDS, and colon cancer; associated with altered splicing and disease progression	[3, 92, 93]
*DHX15*	Core (U2 snRNP helicase)	Point mutations, frameshift	MDS/AML	1–3	Identified in MDS and AML through whole-exome sequencing; DEAH box helicase involved in spliceosome assembly; mutations affect p53 and MDM2 pathways; associated with poor prognosis	[94, 95]
*FUBP1*	RNA binding protein, functions as a general splicing factor	Point mutations, frameshift	Pan-cancer	2–4	Far upstream binding protein 1; identified as driver gene across multiple cancer types through TCGA analysis; functions as a splicing regulator facilitating U2AF2 binding and 3' splice site recognition; associated with altered splicing patterns and immune infiltration; mutations linked to poor prognosis	[4, 5, 11, 96]

Myelodysplastic syndromes (MDS) are a good example of this because more than half of the patients have mutations in genes that are involved in splicing. This makes them one of the most common pathways affected by the disease [12]. Table 1 shows that *SF3B1* mutations are most common in MDS subtypes with ring sideroblasts. *SRSF2* mutations, on the other hand, are quite common in chronic myelomonocytic leukemia (CMML), where they happen in 40–50% of patients. These mutations frequently manifest as early clonal events, either preceding or coinciding with mutations in genes such as *TET2*, *ASXL1*, or *RUNX1*, and are thought to initiate or influence disease progression. [13]. In chronic lymphocytic leukemia (CLL), *SF3B1* mutations are found in about 10–15% of individuals. These mutations are linked to a worse prognosis, accelerated disease progression, and resistance to treatment. Systematic analyses have demonstrated that splice-site-creating mutations constitute a unique category of oncogenic alterations in various cancer types [14]. The high frequency and particular patterns of splicing factor mutations in hematologic malignancies, in contrast to their rarity in most solid tumors, underscore a unique biological reliance in these diseases and create a compelling framework for investigating their downstream molecular impacts.

### Functional and clinical consequences of splicing factor mutations

The molecular mechanisms via which splicing factor mutations facilitate oncogenesis have been thoroughly established at the transcriptome level. These mutations, frequently accumulating in RNA-binding or splicing regulatory domains, operate via neomorphic or gain-of-function processes rather than total loss-of-function [10, 15]. Mutations in splicing factors mostly affect the selection of splice sites, which leads to systematic and non-random alterations in the structure of transcripts that disrupt major physiological pathways [4, 5].

**Fig. 1:**
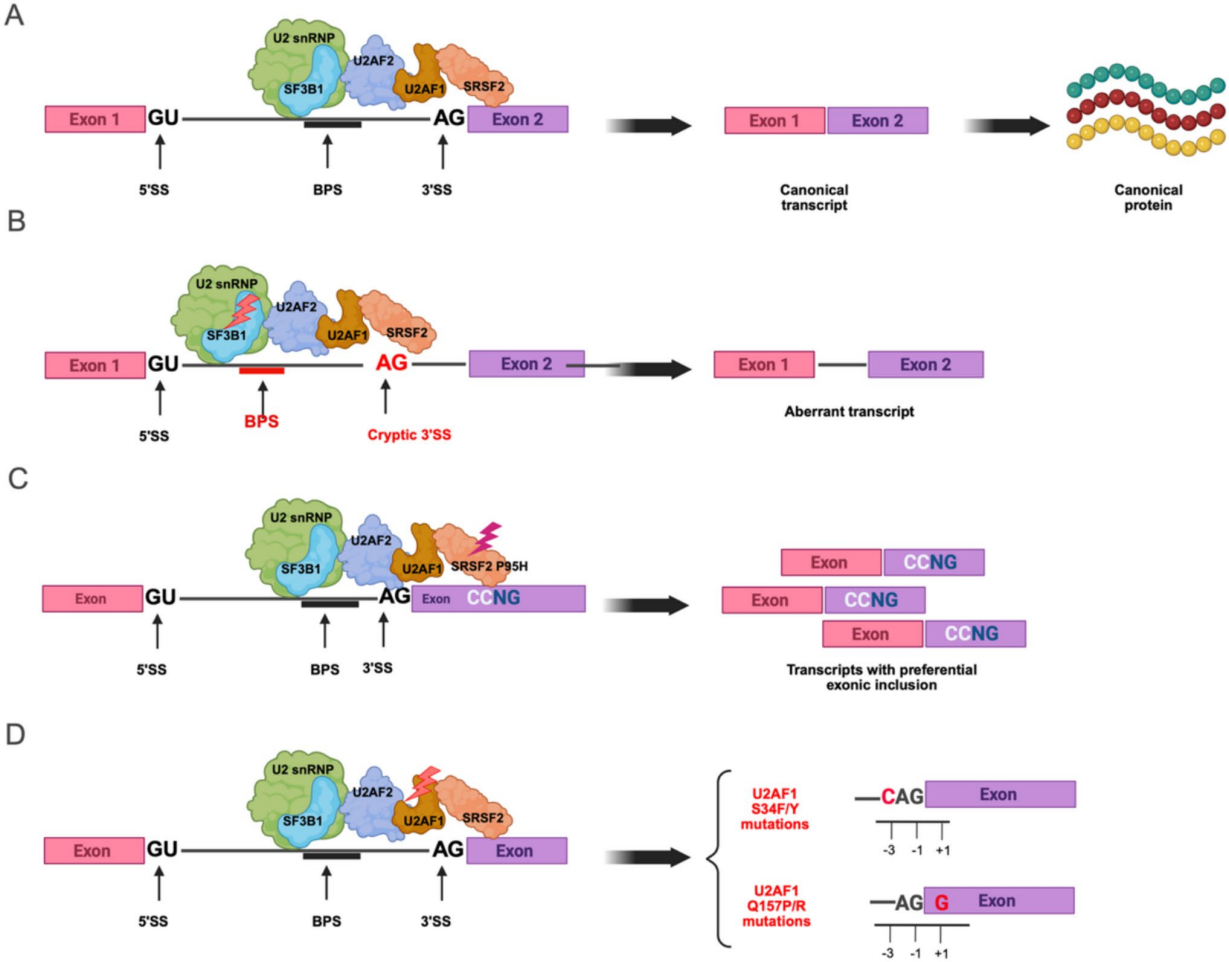
HYPERLINK "sps:id::fig1||locator::gr1||MediaObject::0" Mechanisms of altered 3' splice site recognition by splicing factor mutations. **A** Normal splicing**.** In canonical pre-mRNA splicing, the U2 snRNP complex (containing SF3B1) recognizes the branch point sequence (BPS) adenosine within the intron. U2AF1 and U2AF2 bind to the polypyrimidine tract and AG dinucleotide at the 3' splice site (3'SS), while SRSF2 recognizes exonic splicing enhancer sequences. The spliceosome assembles at the 5' splice site (5'SS, GU dinucleotide), BPS, and 3'SS to catalyze intron removal, generating canonical transcripts that are translated into functional proteins. **B** *SF3B1* mutations. Hotspot mutations in *SF3B1* (e.g., K700E, H662Q, R625C) alter the branch point recognition specificity of the U2 snRNP complex, causing aberrant binding to alternative branch point sequences (BPS') upstream of the canonical site. This altered recognition leads to the usage of cryptic 3' splice sites (cryptic 3'SS), resulting in aberrant or alternative transcripts with altered exon–intron boundaries. These mis-spliced transcripts may encode non-functional proteins, generate neoantigens, or undergo nonsense-mediated decay. **C** Proline 95 mutations in *SRSF2* (P95H/L/R) alter the RNA-binding specificity of this SR protein, increasing its preference for C-rich motifs (CCNG) while decreasing its affinity for G-rich motifs (GGNG). This altered sequence recognition skews exon inclusion decisions: exons containing CCNG motifs experience increased SRSF2 binding and preferential inclusion in mature transcripts, while exons with GGNG motifs show reduced binding and are more frequently skipped. **D** *U2AF1* mutations. Mutations at two distinct hotspots in U2AF1 alter the 3' splice site consensus sequence preferences of this essential splicing factor. *U2AF1* S34F/Y mutations favor the inclusion of cassette exons with a cytosine nucleotide at the "−3" position (CAG motif) relative to the AG dinucleotide, whereas *U2AF1* Q157P/R mutations favor the inclusion of cassette exons with a guanine nucleotide at the " + 1" position (AG G motif). These altered sequence preferences lead to widespread changes in splice site selection, affecting the splicing of genes involved in DNA damage response, epigenetic regulation, and immune signaling pathways. *5'SS*, 5' splice site; *BPS*, branch point sequence; *BPS'*, alternative/aberrant branch point sequence; *3'SS*, 3' splice site; *U2 snRNP*, U2 small nuclear ribonucleoprotein; *U2AF1/2*, U2 auxiliary factor 1/2; *SRSF2*, serine/arginine-rich splicing factor 2; *SF3B1*, splicing factor 3B subunit 1

The most prevalent mutation, *SF3B1* K700E, makes use of cryptic 3′ splice sites that are around 10–30 nucleotides upstream of canonical sites. This leads to frameshifts, premature termination codons (PTCs), and nonsense-mediated mRNA decay (NMD)–sensitive transcripts [16]. *SRSF2* mutations at Proline 95 change how RNA binds to it and how specific it is, making it more likely to recognize C-rich exons than G-rich exons. This changes the patterns of exon inclusion and affects important transcriptional regulators [15]. *U2AF1* mutations (e.g., S34F/Y, Q157R/P) change how the 3′ splice site AG is recognized, which might cause exon skipping or changes in how junctions are used in a sequence-dependent manner [17]. These aberrant splicing events affect many cellular processes, such as DNA repair (e.g., ATR, BRCA1), cell cycle regulation (e.g., CDC25C), apoptosis (e.g., CASP8, BAX), and signal transduction pathways (e.g., JAK-STAT, NF-κB, NOTCH pathways). This could result in proteins that are shorter, have dominant-negative isoforms, or produce neoantigens [18–20]. *U2AF1* mutations have been documented to modify the splicing of RAD51, an essential DNA repair gene, thereby associating splicing dysregulation with DNA damage response in lung adenocarcinomas [21]. The distinct molecular mechanisms by which *SF3B1*, *SRSF2*, and *U2AF1* mutations alter splice site recognition are summarized in Fig. 1. While each mutation affects different steps in the splicing process branch point recognition (SF3B1), exonic splicing enhancer binding (SRSF2), and 3' splice site selection (U2AF1) they converge on a common outcome of widespread splicing dysregulation that disrupts normal cellular functions and drives malignant transformation.

Beyond their direct effects on splicing patterns, *SRSF2* and *U2AF1* mutations induce genomic instability through the accumulation of R-loops (RNA:DNA hybrids), which trigger replication stress and activation of the ATR-Chk1 DNA damage response pathway, leading to double-strand breaks and compromised hematopoietic progenitor function [22]. These R-loops result from impaired transcription pause release, as mutant SRSF2 loses its ability to extract the P-TEFb complex from the 7SK complex, preventing efficient RNA polymerase II elongation and contributing to the genomic instability characteristic of high-risk MDS.

The clinical ramifications of these molecular modifications are significant and influence disease biology and treatment efficacy. In myelodysplastic syndromes, splicing factor mutations manifest in up to 80% of patients, frequently as founder events that delineate disease subgroups and influence clonal architecture [23]. *SF3B1* mutations are related with myelodysplastic syndromes characterized by ring sideroblasts and typically favorable prognoses, whereas *SRSF2* and *U2AF1* mutations correlate with unfavorable characteristics and progression to secondary acute myeloid leukemia. [24]. In chronic lymphocytic leukemia, *SF3B1* mutations are associated with increased genomic instability, inferior prognosis, and shorter time to first treatment [25]. These clinical observations support the hypothesis that splicing dysregulation disrupts transcript fidelity and influences tumor evolution, therapeutic resistance, and disease heterogeneity, thereby facilitating the exploration of how these consequences go beyond the transcriptome to affect protein function via post-translational modification networks.

## Post-translational modification disruption as a downstream consequence of splicing dysregulation

### Overview of post-translational modifications and their role in cancer

The move from viewing splicing mutations as merely transcriptomic events to realizing their broader impact on cellular function requires analyzing their impacts on post-translational modification (PTM) networks. PTMs are covalent enzymatic changes that happen to proteins after they are made. They are extremely essential for controlling almost every part of how proteins perform their functions [26]. Ubiquitination, phosphorylation, acetylation, methylation, SUMOylation, ISGylation, and other modifications control important biological processes that include protein stability, folding, subcellular localization, complex formation, and enzymatic activity [27].

The dynamic nature and reversibility of many PTMs allow cells to respond rapidly to internal and external signals, ensuring homeostatic control of growth, differentiation, metabolism, and stress responses [28]. Ubiquitination, for instance, usually marks proteins for the proteasome to break down, phosphorylation changes the way signal transduction cascades work, and acetylation controls gene expression and chromatin dynamics [29, 30]. In cancer, PTM networks are often compromised to facilitate malignant transformation and progression via mutations in PTM enzymes, dysregulated expression, or mislocalization of PTM machinery [31].

The therapeutic importance of PTM pathways in cancer is extensively documented, as evidenced by the effectiveness of proteasome inhibitors in multiple myeloma, HDAC inhibitors in hematologic malignancies, and kinase inhibitors in various solid tumors [32, 33]. This underscores the potential of PTM-modulating agents to function as both precision therapies and combinatorial strategies, particularly in cancers characterized by disrupted PTM balance. The growing evidence that splicing dysregulation may indirectly alter PTM landscapes signifies a new aspect of tumor biology with considerable implications for both mechanistic comprehension and therapeutic strategies. Altered RNA processing is an accelerator for tumor pathogenesis and an attractive target for therapeutic targeting [34].

### Mechanisms linking splicing dysregulation to PTM disruption

The link between splicing mutations and PTM disruption works through a number of interconnected mechanisms that spread the effects of wrong RNA processing into the proteomic landscape [15, 16]. Table 2 gives an overview of the main PTM pathways that are interrupted up by splicing mutations. This helps us understand these intricate interactions. These disruptions can be grouped into three main pathways based on the molecular mechanisms where the cellular PTM landscape is altered.

**Table 2:** Post-Translational Modification Pathways Disrupted by Splicing Mutations

PTM Pathway	Key Enzymes/Substrates Affected	Splicing Mutation	Functional Consequence	Therapeutic Target	References
Ubiquitination	UBA1 (E1 enzyme)	*SF3B1*-K700E	Reduced protein levels, proteasome dysfunction	TAK-243 (UBA1 inhibitor)	[37]
ISGylation	UBA7 (E1 enzyme)	*SF3B1*-K700E	Impaired immune signaling, reduced ISG15 conjugation	ISGylation modulators	[38]
Iron homeostasis	ABCB7 (iron transporter)	*SF3B1*-K700E	Iron-sulfur cluster biogenesis defects	Iron chelators	[42]
Phosphorylation	PP2A-B56α (phosphatase)	*SF3B1*	MYC stabilization, oncogenic signaling	PP2A activators	[40, 48]
Chromatin regulation	EZH2 (methyltransferase)	*SRSF2*-P95H/L/R	Reduced histone methylation, epigenetic dysregulation	EZH2 inhibitors	[15]
Exitron targets	Multiple cancer drivers	Pan-splicing mutations	Protein domain loss, altered PTM sites	Targeted therapies	[50, 53]
NMD substrates	PTM enzyme transcripts	*SF3B1*, *U2AF1*	Systematic PTM enzyme depletion	NMD inhibitors	[54]

#### Enzyme-level PTM disruption: direct targeting of modification machinery

This mechanism directly mis-splices genes that code for PTM-regulating enzymes ending with changes the cellular modification machinery in either a quantitative or qualitative way. Missplicing can lead to frameshifts, exon skipping, or retained introns, resulting in nonfunctional or unstable transcripts, many of which are subject to nonsense-mediated decay (NMD [35]. The enzymatic machinery necessary for sustaining PTM homeostasis is either quantitatively diminished or qualitatively modified, resulting in subsequent impacts on protein stability, degradation, signaling fidelity, and chromatin state. Indeed, a systematic analysis of PTM diversification across isoforms has revealed that a significant percentage of PTMs are excluded from at least one isoform, highlighting the widespread impact of splicing on the PTM landscape [36].

The most convincing proof of this mechanism comes from studies that show that *SF3B1* K700E mutations cause UBA1, the important E1 ubiquitin-activating enzyme, to be systematically mis-spliced [37]. This mis-splicing creates a shortened version of UBA1 (UBA1ms) that is missing important enzymatic domains. This makes the protein unstable and lowers the total amount of UBA1. The functional outcome is a global decrease in the ability to ubiquitinate, which makes cells more susceptible to UBA1 inhibition by small molecules like TAK-243. In addition to these findings, mutations in *SF3B1* also lead to the downregulation of UBA7, the E1 enzyme responsible for ISGylation [38], This makes ISG15 conjugation less effective and interferon signaling less potent. This shows that splicing errors can affect more than one PTM pathway at the same time in the same cell.

Beyond E1 ubiquitin-activating enzymes, splicing mutations dysregulate the broader PTM enzyme machinery including E2-conjugating enzymes, E3 ubiquitin ligases, and SUMO E3 ligases (PIAS family), which collectively determine substrate specificity and ubiquitin chain topology in cellular ubiquitination networks. Splicing factor mutations also impact kinase signaling cascades by affecting the splicing of genes encoding DNA damage response kinases (ATM, ATR, CHK2), cell cycle kinases (CDKs, cyclins), and receptor tyrosine kinases, thereby disrupting checkpoint control and proliferation signals critical for maintaining genomic stability. Phosphatase dysregulation represents a particularly well-characterized consequence of splicing mutations, exemplified by SF3B1-mediated mis-splicing of PPP2R5A (encoding PP2A-B56α), which leads to loss of this critical tumor suppressor phosphatase and hyperphosphorylation of oncoproteins such as MYC. While the systematic dysregulation of E2/E3 ligase and kinase genes in splicing-mutant cancers has been documented through transcriptomic and proteomic analyses, the specific mechanisms by which individual splicing factor mutations alter the splicing patterns of these genes remain incompletely understood and warrant comprehensive functional studies to decipher how splicing dysregulation of ubiquitin ligases and kinases contributes to the oncogenic phenotype [2, 39–41].

In addition to ubiquitin-like modifications, *SF3B1* mutations cause ABCB7 to be mis-spliced using the wrong 3′ splice site. This causes nonsense-mediated decay and lowers the levels of *ABCB7* mRNA and protein [42]. Since ABCB7 is subject to iron-dependent post-translational regulation, its loss disrupts both iron homeostasis and the cellular capacity for iron-sulfur cluster biogenesis, illustrating how splicing defects can indirectly affect PTM-dependent metabolic processes. These findings collectively demonstrate that mutations in splicing factors consistently impact the enzymatic machinery governing post-translational modification regulation, leading to pervasive deficiencies in cellular modification capacity that fundamentally disrupt protein homeostasis.

#### Substrate-level PTM disruption: loss of modification sites and regulatory domains

Exon skipping, cryptic splice site usage, or isoform switching are all ways that this mechanism changes PTM substrates themselves by removing or changing important PTM recognition motifs [18, 19, 43]. Mutant splicing factors can cause alternative splicing, which makes protein isoforms with changed or missing PTM recognition motifs, like phosphorylation or ubiquitination consensus sequences. For example, skipping one exon could remove a phosphorylation site that is needed for activation or a lysine residue that is needed for ubiquitin conjugation. These changes make proteins less likely to bind to substrates, interfere with signaling cascades, or make proteins resistant to degradation, thereby facilitating oncogenic stabilization or pathway rewiring. Crucially, these aberrant splicing events can also lead to the generation of neoantigens, which are novel protein sequences that can be recognized by the immune system [44, 45].

*U2AF1* mutations (S34F/Y) illustrate this mechanism by systematically modifying splice site recognition, resulting in enhanced exon skipping in roughly 77% of significantly altered transcripts [43]. This widespread exon skipping affects genes encoding proteins with critical PTM sites, including kinases, phosphatases, and ubiquitin pathway components, where the loss of exons containing phosphorylation consensus sequences or ubiquitination motifs fundamentally alters post-translational regulation. In the same way, *SF3B1* K700E mutations make it more likely that cryptic 3′ splice sites that are 10–30 nucleotides upstream of canonical sites will be used. This often leads to frameshift mutations that affect downstream PTM domains [23, 46]. This systematic change in splice site selection affects hundreds of transcripts. Some proteins, like MZB1, PHGDH, ABCB7, and SEPT6, are affected by nonsense-mediated decay, which leads to a decrease in protein levels in mutant SF3B1 cells.

The effect on chromatin regulation is another important example. Mutations in *SRSF2* P95H/L/R change the protein's RNA-binding specificity, which makes it more likely to skip exons with certain sequence motifs [47]. One very important effect is that a poison exon in EZH2 that goes through nonsense-mediated decay causes *SRSF2*-mutant cells to make less EZH2 protein. The extensive post-translational regulation of EZH2 through phosphorylation and ubiquitination that controls its histone methyltransferase activity and protein stability suggests the loss of EZH2 through incorrect splicing fundamentally disrupts chromatin modification networks and epigenetic regulation. Additionally, mutations in *SF3B1* cause PPP2R5A (which encodes PP2A-B56α) to be mis-spliced in a systematic way, which causes the loss of this important tumor suppressor phosphatase [40, 48]. PP2A-B56α normally removes phosphate groups from MYC at certain serine/threonine residues, which helps break down MYC. However, when it is lost through mis-splicing, MYC becomes hyperphosphorylated and stabilized. These examples show that splicing mutations that disrupt substrate-level PTM create an environment where important regulatory proteins lose their normal ways of controlling post-translational events, which makes it easier for cancer to spread.

#### Proteome-wide PTM disruption: exitron splicing and cryptic transcript generation

This mechanism exemplifies the most systematic form of post-translational modification (PTM) disruption, wherein aberrant splicing machinery induces extensive modifications in protein domains via exitron splicing and the generation of cryptic transcripts. These occurrences fundamentally alter protein structure, resulting in internally deleted proteins or truncated variants that lack critical PTM regulatory domains. Exitron splicing makes cryptic introns inside protein-coding exons, which makes proteins that are missing parts that change protein domains, disordered regions, and different post-translational modification sites [49]. Pan-cancer analysis shows that exitron splicing has an effect on 63% of human coding genes, and 95% of these events are unique to tumors [50], and they show a pattern that is mutually exclusive with somatic mutations. This suggests that exitron splicing is an alternative way to change cancer driver genes.

The systematic aspect of exitron splicing is demonstrated by its impact on cancer driver genes, as it alters both established and novel cancer driver genes, leading to gain- or loss-of-function modifications that promote tumor progression [51, 52]. These changes often affect protein domains that are important for regulating PTMs, such as kinase domains, phosphatase domains, and ubiquitin-binding regions. This can lead to proteins that lose important PTM sites or gain strange modification patterns. The resulting protein diversity serves as a driving force for creating proteomic complexity, with exitron-containing and exitron-spliced isoforms contributing significantly to altered PTM networks [53]. This diversity particularly affects PTM regulation, as exitron splicing can create protein variants with novel combinations of modification sites, altered domain architectures, and changed regulatory properties.

The therapeutic ramifications of this mechanism are illuminated by the susceptibility caused by cryptic transcript generation. Mutations in splicing factors create hundreds of abnormal transcripts with premature termination codons as a result of cryptic splice site usage and exitron inclusion. These transcripts are more likely to be targeted for nonsense-mediated decay, which causes a systematic loss of PTM enzymes like ubiquitin ligases, kinases, and phosphatases [54]. The reliance of splicing-mutant cells on handling this load of hidden transcripts leads to therapeutic weaknesses. In these cases, NMD inhibition accumulates cancer cells with harmful proteins while leaving normal cells with fewer NMD substrates alone. When UPF1 inhibitors are used on cancer cells with *SF3B1* or *U2AF1* mutations, they build up abnormal proteins, such as truncated PTM enzymes that can act as dominant-negative regulators. This causes a chain reaction of PTM dysfunction that leads to the death of the cells. This proteome-wide disruption mechanism thus represents both the most comprehensive form of PTM network alteration and a promising avenue for therapeutic intervention in splicing-mutant cancers.

### Convergence of PTM disruption mechanisms and cellular transformation

These three complementary mechanisms of PTM disruption converge to create a complex cellular environment where normal post-translational regulation is fundamentally altered. The inability to ubiquitinate due to enzyme mis-splicing, along with changes in phosphorylation patterns from substrate modification and the systematic loss of PTM regulators through cryptic transcript generation, makes cells that are both more resistant to apoptosis and more sensitive to targeted therapies. Disruption of ubiquitination systems is the most convincing example of how splicing mutations can create specific therapeutic vulnerabilities while also affecting immune function. This is because splicing mutations affect many different PTM pathways.

In addition to ubiquitination and ISGylation, splicing mutations influence various other post-translational modification pathways, resulting in considerable functional impacts [55]. Phosphorylation networks are significantly impaired, with notable instances such as the SF3B1-mediated aberrant splicing of PP2A-B56α resulting in MYC stabilization and oncogenic signaling [40, 48], and changes in DNA damage response kinases like ATM and ATR that make instability at the genome level [18, 19, 56, 57]. Changes to HDAC complexes, especially in cells with *SRSF2* and *U2AF1* mutations, disrupt the normal acetylation pathways, which affect chromatin remodeling regulation [58].

The convergence of PTM pathway disruptions has profound implications for immune recognition and tumor surveillance, as post-translational modifications critically regulate antigen presentation and immune cell activation. Splicing-induced PTM loss fundamentally alters the immunopeptidome the repertoire of peptides presented on major histocompatibility complex (MHC) molecules through multiple mechanisms. First, dysregulation of ubiquitination and phosphorylation enzymes impairs the proteolytic processing of proteins required for generating MHC-binding peptides, reducing the diversity and abundance of canonical self-antigens presented to T cells. Second, aberrant splicing generates cryptic transcripts encoding truncated or internally deleted proteins that, when processed, generate novel peptide epitopes with altered PTM patterns that can escape immune recognition or, conversely, generate immunogenic neoantigens. Third, splicing-induced loss of PTM enzymes directly affects the PTM status of MHC molecules themselves and immune checkpoint proteins (PD-L1, CTLA-4, LAG-3), altering their stability, trafficking, and immunological function. Dysregulation of kinase and phosphatase splicing particularly impacts T cell receptor (TCR) signaling, NF-κB pathway activation, and interferon-γ production, thereby impairing both CD8 + T cell-mediated cytotoxicity and CD4 + T helper cell differentiation. These mechanistic links between splicing-induced PTM disruption and immune dysfunction create a multi-layered immune evasion strategy, where tumors simultaneously reduce the presentation of canonical antigens while evading recognition of aberrant neoantigens through altered checkpoint protein PTM status [2, 59–61].

The convergence of various PTM pathway disruptions leads to a complex environment in which tumors can simultaneously acquire therapeutic vulnerabilities while circumventing standard cellular regulatory mechanisms. This multi-pathway disruption shows that *SF3B1* and other splicing factor mutations don't just change RNA; they also change proteomic and PTM landscapes in ways that add more fitness to cancer cells. The cumulative effect goes far beyond just making one protein not work; it changes whole signaling networks, especially those that control how the immune system recognizes and watches for tumors [62].

Beyond immune evasion, splicing-induced PTM loss creates profound proteostasis imbalance through endoplasmic reticulum (ER) stress and unfolded protein response (UPR) dysregulation [63]. The systematic loss of ubiquitination and acetylation enzymes impairs the quality control mechanisms that normally degrade misfolded proteins, leading to accumulation of aberrant protein isoforms generated by cryptic splicing events. These truncated or internally deleted proteins, which often lack critical PTM recognition motifs required for proper folding, aggregation, or degradation, accumulate in the ER lumen and trigger ER stress. The resulting unfolded protein response (UPR), normally a protective mechanism involving PERK, IRE1α, and ATF6 signaling, becomes chronically activated in splicing-mutant cells, leading to sustained phosphorylation of eIF2α and translational attenuation. Paradoxically, while this chronic UPR initially suppresses protein synthesis to reduce ER burden, it also promotes the expression of pro-survival genes and metabolic reprogramming that support cancer cell survival and proliferation. Furthermore, splicing mutations that affect genes encoding chaperone proteins (HSP90, BiP, GRP78) and proteasome components exacerbate proteostasis imbalance by reducing the capacity to process misfolded proteins. The convergence of PTM enzyme loss, aberrant protein accumulation, and chronic UPR activation creates a state of proteostasis crisis that paradoxically enhances cellular transformation by selecting for cancer cells capable of surviving under conditions of extreme protein misfolding stress. This proteostasis imbalance represents both a vulnerability as demonstrated by the efficacy of proteasome inhibitors and HSP90 inhibitors in splicing-mutant cancers and a driving force for malignant transformation through selection of cells with enhanced stress tolerance and metabolic flexibility [64–67].

This proteome-wide disruption of PTM networks signifies a previously overlooked mechanism through which splicing mutations facilitate oncogenesis, extending beyond their recognized function in transcript diversity to elucidate their essential influence on protein functionality and cellular signaling networks that regulate cancer progression and immune surveillance. The systematic nature of these alterations, along with their therapeutic weaknesses, makes PTM disruption a key effect of splicing dysfunction in cancer biology. Comprehending the specific effects of these PTM alterations on immune system function yields essential insights into the mechanisms through which splicing-mutant tumors evade immune surveillance and acquire resistance to immunotherapy.

## Splicing mutations and the immune landscape

### Splicing-mediated immune evasion and tumor surveillance

The intersection of splicing mutations with immune system function represents a rapidly emerging area of research with profound implications for cancer immunotherapy [68]. Disruption of PTM networks through splicing mutations transcends cellular housekeeping functions, fundamentally altering tumor-immune system interactions. This link is especially important because post-translational modifications are very important for immune signaling, antigen presentation, and how immune cells function.

Aberrant RNA splicing can produce numerous neoantigens, potentially surpassing those generated by somatic mutations, which may either enhance or suppress the tumor immune response. [69]. Recent studies have revealed that tumor-wide RNA splicing aberrations can generate a class of 'public neoantigens' that are shared across diverse cancer types, providing a promising avenue for broadly applicable immunotherapies [65]. Tumor cells can exploit alternative splicing to modify the expression of their surface antigens, thereby impairing the immune system's ability to recognize and eliminate them. For instance, the secretion of a soluble splice variant of PD-L1 (scePD-L1) can interfere with the PD-1/PD-L1 signaling pathway, aiding tumor cells in evading immune attack [70]. Similarly, soluble CTLA-4 (sCTLA-4) can block CD8 + T-cell activation [71–74], while tumor-produced splice variants can influence the tumor microenvironment by promoting immunosuppressive macrophage polarization [75].

On the other hand, aberrant splicing can make tumors more immunogenic by creating new splice junctions that make tumor-specific peptides presented by MHC molecules. This could change "cold" tumors into "hot" tumors, making them more likely to respond to immunotherapy. [76–78]. The identification of cognate T-cell receptors (TCRs) that recognize these splicing-derived neoantigens has opened up new possibilities for engineered T-cell therapies [44, 45]. Additionally, aberrant splicing can cause double-stranded RNA (dsRNA) to build up in the cytoplasm. This activates antiviral signaling pathways and boosts immune surveillance by activating dendritic cells, T cells, and improving tumor immune surveillance.

### Impact of splicing mutations on immune cell function

Splicing mutations not only directly affect tumor cells, but they also have major impacts on how immune cells work in the tumor microenvironment [29, 30, 109]. Table 3 summarizes the main immune-related effects of splicing mutations, showing how many different ways that RNA processing can go wrong and cause problems with the immune system.

**Table 3:** Immune-related consequences of splicing alterations

Immune Process	Splicing Target	Mutation	Consequence	Therapeutic Implication	References
Neoantigen Generation	Multiple genes	*SF3B1, SRSF2, U2AF1*	Increased immunogenicity	Enhanced immunotherapy response	[97–99]
T-regulatory Cell Function	Anapc13	*SF3B1*-K700E	Impaired immune suppression	Immune surveillance restoration	[79, 100, 101]
Interferon Signaling	UBA1, UBA7	*SF3B1*	Reduced antiviral response	Immune evasion	[37, 38]
Checkpoint Regulation	PD-L1, CTLA-4	Splicing aberration	Soluble inhibitory variants	Combination immunotherapy	[71, 72, 76, 77, 102, 103]
Macrophage Polarization	DCLK1	Splicing variants	M1 to M2 shift	Immunosuppression regulation	[104, 105]
Cytokine Production	MyD88, TLR4, NLRP3	Splicing aberration	Altered inflammatory response	Immune modulation	[106–108]

A particularly notable example arises from investigations of the *SF3B1*-K700E mutation in regulatory T cells (Tregs), which causes impaired Treg differentiation and function, culminating in spontaneous autoimmune phenotypes [79]. This disorder is a result of the abnormal splicing of the *Anapc13* gene, which hinders Treg-mediated immune suppression. In aged mice with this mutation, impaired immune surveillance accelerates the proliferation of acute myeloid leukemia, underscoring the essential function of splicing fidelity in preserving immune homeostasis and inhibiting tumor advancement.

Alternative splicing is commonly observed in immune cells, with unique splicing events taking place in macrophages and T cells upon activation [68]. These splicing events control important immune signaling pathways, such as those that involve MyD88, TLR4, and NLRP3. This changes how immune cells work and how they respond to inflammation. Cancer-associated mutations can mess up these splicing events, which can have big effects on the immune system's ability to fight tumors. This creates a complicated relationship between splicing problems, PTM problems, and immune evasion. The combination of splicing mutations, PTM disruption, and immune dysfunction makes it possible for tumors to avoid immune surveillance while also becoming more sensitive to targeted therapies. This opens the door for new treatments that take advantage of these weaknesses.

## Therapeutic implications and future directions

### Current therapeutic strategies and clinical trials

The increasing recognition of the complex relationships among splicing mutations, post-translational modifications (PTMs), and the immune system has unveiled novel therapeutic opportunities that surpass conventional strategies focused solely on splicing machinery [80, 81]. The finding that *SF3B1*-mutant cells are specifically susceptible to UBA1 inhibition due to UBA1 mis-splicing exemplifies precision therapy for splicing-mutant cancers. Likewise, the downregulation of UBA7 in SF3B1-mutant MDS indicates that targeting the ISGylation pathway may constitute a feasible therapeutic approach. Table 4 presents a thorough summary of ongoing clinical trials and therapeutic strategies aimed at splicing-related vulnerabilities, illustrating the application of mechanistic insights to clinical contexts. TAK-243 is a UBA1 inhibitor that is currently in Phase I trials for advanced solid tumors and lymphomas (NCT06223542). It directly targets the weakness caused by SF3B1-mediated UBA1 mis-splicing.

**Table 4:** Current Clinical Trials Targeting Splicing-Related Vulnerabilities

Drug/Compound	Target	Mechanism	Cancer Type	Trial Phase	ClinicalTrials.gov ID
TAK-243	UBA1	Ubiquitin-activating enzyme inhibitor	Advanced solid tumors, lymphomas	Phase I	NCT06223542
H3B-8800	SF3B1	Splicing modulator	Myeloid malignancies	Phase I	NCT02841540
E7820	RBM39	Splicing factor degrader	Myeloid malignancies with SF mutations	Phase II	NCT05024994
Tasisulam	RBM39	Splicing factor degrader	Advanced solid tumors	Phase I	NCT01284335
Tasisulam	RBM39	Splicing factor degrader	Sarcoma	Phase II	NCT00490451
Tasisulam	RBM39	Splicing factor degrader	Metastatic melanoma	Phase III	NCT01006252
E7070	RBM39	Splicing factor degrader	Relapsed or Refractory Acute Myeloid Leukemia and High-Risk Myelodysplastic Syndrome	Phase II	NCT01692197
E7070	RBM39	Splicing factor degrader	Metastatic melanoma	Phase II	NCT00014625
PF-06939999	PRMT5	Splicing factors methylation inhibition	Metastatic solid tumors	Phase I	NCT03854227
JNJ-64	PRMT5	Splicing factors methylation inhibition	Non-Hodgkin lymphoma and solid tumors	Phase I	NCT03573310
MRTX1719	PRMT5	Splicing factors methylation inhibition	Advanced solid tumors	Phase I/II	NCT05245500
AMG 193	PRMT5	Splicing factors methylation inhibition	Advanced solid tumors	Phase I/II	NCT05094336
SM08502	SRSF	SRSF phosphorylation inhibitor	Advanced solid tumors	Phase I	NCT03355066
Spliceostatin A	SF3B1	Spliceosome inhibitor	Preclinical	-	-
Pladienolide B	SF3B1	Spliceosome inhibitor	Preclinical	-	-

The immunomodulatory effects of splicing mutations also present significant therapeutic opportunities [82]. The creation of neoantigens through incorrect splicing can be used to make immune checkpoint inhibitors function more effectively. These neoantigens derived from mis-splicing can provoke T cell responses and serve as potential targets for immunotherapy in splicing factor mutant leukemias [45]. Therapeutic strategies designed to modify splicing to enhance neoantigen presentation or activate antiviral signaling pathways may transform immunologically "cold" tumors into "hot" tumors, thereby increasing their susceptibility to immunotherapy.

### Future research directions and challenges

Future investigations must concentrate on several critical domains to fully exploit the therapeutic potential of targeting splicing-induced post-translational modifications and immune dysregulation. A more thorough comprehension of the array of post-translational modifications (PTMs) influenced by various splicing mutations is essential, necessitating sophisticated proteomic methodologies to detect and measure PTM alterations in splicing-mutant cells. Moreover, the specific mechanisms by which splicing-induced PTM modifications influence the immune system require clarification through comprehensive investigations of PTM impacts on immune cell functionality and tumor-immune interactions.

Creating new treatments that focus on the vulnerabilities caused by splicing-driven PTM and immune dysregulation is essential. This encompasses the formulation of combination therapies that concurrently address splicing vulnerabilities and augment immune responses, alongside the advancement of biomarkers to identify patients most likely to benefit from these strategies. Additionally, it will be important to know how splicing-driven changes change over time and how they change as the disease gets worse to find the best time and order for treatment.

## Conclusion

**Fig. 2:**
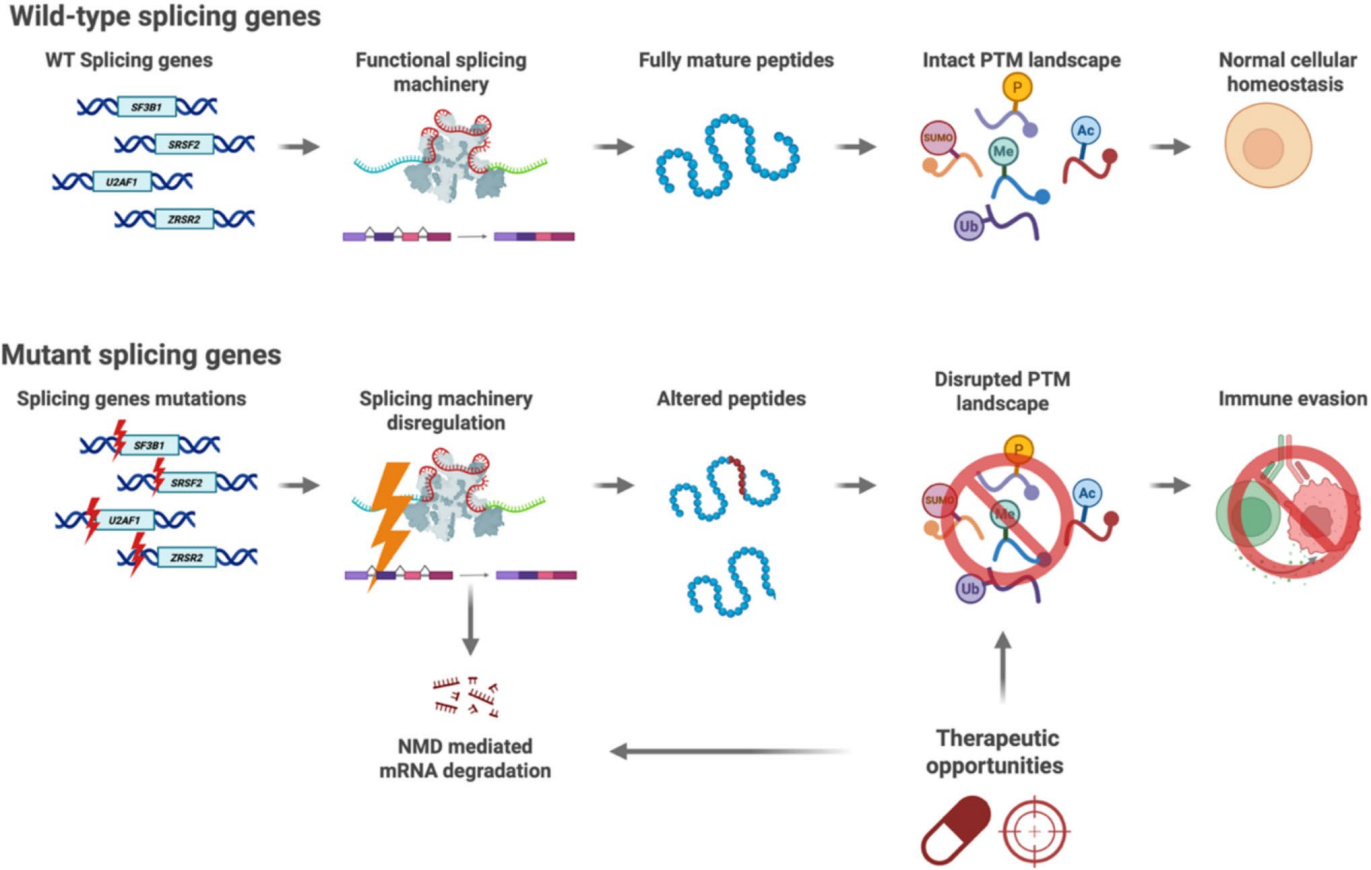
Mutations in splicing factors cause problems with post-translational modification and help cancer cells avoid the immune system. Upper Panel (Wild-type splicing genes): Functional splicing machinery keeps cells in a normal state of homeostasis. Wild-type splicing factors (SF3B1, SRSF2, U2AF1, ZRSR2) make sure that pre-mRNA is processed correctly, which leads to completely mature peptides with intact post-translational modification (PTM) landscapes that include phosphorylation (P), ubiquitination (Ub), acetylation (Ac), methylation (Me), and SUMOylation (SUMO). This keeps the cells' normal balance and immune system surveillance. Lower Panel (Mutant splicing genes): Mutations in splicing factors cause the splicing machinery to work incorrectly, which leads to the production of altered peptides through incorrect splicing events. This causes a defective PTM landscape, where regular protein modifications are lost or altered. The cascade causes immune evasion where tumor cells (red) escape immune surveillance by avoiding immune cells (green). Aberrant transcripts are also subject to nonsense-mediated degradation (NMD), which diminishes the quantity of functional proteins. Therapeutic Options: The changed PTM landscape and splicing dependencies make weaknesses that can be used for therapeutic purposes with targeted drugs and precision medicine methods. *P*, phosphorylation; *Ub*, ubiquitination; *Ac*, acetylation; *Me*, methylation; *SUMO*, SUMOylation; *NMD*, nonsense-mediated decay

The integration of RNA splicing research, post-translational modification biology, and tumor immunology has uncovered a complex and interconnected network of molecular events that facilitate tumorigenesis via previously unrecognized mechanisms. Splicing mutations, previously recognized primarily for their transcriptomic consequences, are now understood to have substantial downstream effects on the proteome and immune landscape by disrupting post-translational modification networks. Figure 2 shows that the cascade from splicing factor mutations to post-translational network disruption and finally to immune evasion is a major shift in how we think about how RNA processing errors contribute to cancer via altering the proteome landscape.

The dysregulation of PTM pathways, particularly ubiquitination systems and other critical modification networks, represents a key mechanism by which splicing mutations contribute to cancer development and progression. At the same time, changing the way immune cells work and watch over tumors gives us new information about how tumors with splicing mutations become more fit by changing the pathways that control the immune system and watch over tumors. This new knowledge opens up a lot of doors for creating new ways to diagnose and treat splicing-mutant cancers by focusing on their specific weaknesses.

The therapeutic landscape is rapidly evolving, with promising clinical trials targeting splicing-related vulnerabilities and the potential for combination approaches that simultaneously exploit metabolic dependencies and enhance immune responses. As we learn more about these complicated interactions, the combination of splicing biology, proteomics, and immunology holds the promise of creating new ways to treat cancer that go beyond traditional methods and take into account the full complexity of tumor biology.

## Data Availability

Not applicable.
